# Impact of nanoparticles on human health and disease

**DOI:** 10.3126/nje.v13i4.61245

**Published:** 2023-12-31

**Authors:** Bedanta Roy, Karthikeyan Murugesan, Maghimaa Mathanmohun, Brijesh Sathian

**Affiliations:** 1 Associate Professor, Department of Physiology, Faculty of Medicine, Quest International University, No. 227, Plaza Teh Teng Seng (Level 2), Jalan Raja Permaisuri Bainun, 30250 Ipoh, Perak Darul Ridzuan, Malaysia; 2 Associate Professor, Department of Microbiology, Faculty of Medicine, Quest International University, No. 227, Plaza Teh Teng Seng (Level 2), Jalan Raja Permaisuri Bainun, 30250 Ipoh, Perak Darul Ridzuan, Malaysia; 3 Assistant Professor, Department of Microbiology, Muthayammal College of Arts and Science, Rasipuram 637408, Tamil Nadu, India; 4 Geriatrics and long term care department, Rumailah Hospital, Hamad Medical Corporation, Doha, Qatar

**Keywords:** Cellular, effect, nanoparticles, signalling, toxicity

## Abstract

Nanoparticles (NPs) are small particles with a surface area ranging from 1 to 100 nm in diameter that are rampantly used in different fields, e.g., medicine, engineering, and others. Because of their unique properties, such as their tiny size, magnetic properties, quantum size effects, and macroscopic quantum tunnelling effects, they are crucial for a wide range of potential applications. NPs play a significant role in the treatment of vascular disorders, the production of vaccines, and the development of drug carriers for diverse therapies due to their bioavailability, targeting ability, and efficacy. However, significant risks to the environment and health are also associated with it. NPs cause necrotic plasma membrane rupture or apoptosis, which leads to cell death. NPs interfere with cell signalling, endosomal membranes, and organelles like the nucleus or mitochondria, affecting their function. NPs cause autophagic cell death, which causes a stress response and sterile inflammation. The primary routes for the entry of NPs into the human body are inhalation, ingestion, and skin contact. NPs accumulate in the respiratory system based on their size, shape, and surface properties. NPs can cause lung inflammation and fibrosis, disrupt the endocrine system by attaching to hormone receptors, and produce reactive oxygen species (ROS) associated with DNA damage, oligospermia, and male infertility. Carcinogenic properties of NPs cause mutations, apoptosis, and inflammatory responses. Collaborative research between ecologists and epidemiologists may enlighten ways to reduce the harmful effects of NPs.

## Background

### Advantages of NPs

Malaria, TB, and HIV are global public health concerns, and nanosized carriers can help overcome challenges such as limited bioavailability, drug buildup, and low patient adherence [1]. Because nanoparticles have distinct optical and electrochemical properties, they are critical for the diagnosis and treatment of vascular disorders. They can detect biomarkers in plasma, serum, and urine, and can be employed as contrast agents in illnesses to visualize pathological changes. However, the use of nanoparticles in vascular disorders is now limited to basic research, with safety and toxicity being major concerns [2]. Cardiovascular disease (CVD) is the primary cause of death worldwide, and nanomedical innovations aim to enhance patient outcomes through revolutionary treatments, imaging agents, and ex vivo diagnostics [3]. Lipid nanoparticles have been employed in the production of COVID-19 mRNA vaccines, which were the first mRNA vaccines approved for emergency use by UK regulators [4]. Lipid nanoparticles can be used as a platform technology, allowing a single nanoparticle to carry several nucleic acids. Nanoparticle (NP) designs optimized for therapeutic delivery and overcoming biological obstacles observed across patient populations and diseases can improve patient stratification approaches, broaden access to precision medicine, and boost the overall therapeutic efficacy [5]. As more advanced NP designs have been investigated, this discovery may have an impact on the future rational design of drug carriers for diverse therapies, both personalized and generic, benefiting a variety of cargos, such as small molecules, nucleotides, and proteins. Nanomedicines outperform traditional pharmaceuticals in terms of drug bioavailability, targeting ability, efficacy, and safety [6]. However, owing to the incompatibility between animal models and humans, clinical usage remains limited. Improving blood circulation, biodistribution, and tissue accessibility may improve the clinical translation of nanomedicine. Understanding the mechanisms impacting these features as well as overcoming biological barriers can aid in the development of nanomedicines with higher delivery efficiency. Bioengineering techniques can improve tissue targeting and optimize nanomedicine designs.

### Disadvantages of NPs

NPs have a large surface area, which enables them to interact with molecules, catalyse chemical reactions, and play a vital role in toxicity. NPs reactions and toxicity are dependent on their specific characteristic features. Reduced NP size enhances the dispersion of silver in tissues and amplifies its toxicity to the liver and bile ducts [7]. The dispersion and buildup of NPs are contingent upon their surface charge and degree of aggregation. NPs exhibit unique characteristics such as the tiny size effect, distinctive magnetic properties, the quantum size effect, and the macroscopic quantum tunnelling effect. These traits make them extensively used in many disciplines. So, there is rampant use of NPs in electronic devices, the invention of new drugs, and the development of materials for food testing and pollution monitoring [8].

Nevertheless, there are apprehensions regarding the potential physiological and environmental risks associated with the manufacture, processing, utilisation, and recycling of NPs. Certain NPs possess exceptional features that interfere with the immune system of the human body. Contamination associated with NPs commonly leads to respiratory symptoms and inflammatory reactions [9]. The risks that NPs pose to human health and their toxicokinetic effects on both people and the environment are currently under investigation. In order to reduce social risks and enhance sustainability, it is necessary to foster interdisciplinary collaboration and apply risk statute theories.

### Mechanism of NP toxicity in mammalian cells

High concentrations of NPs are toxic to cellular structures, potentially leading to cell death through necrotic plasma membrane rupture or apoptosis. Surfactant molecules associated with NP preparations can disrupt the plasma membrane, and cellular structures and hinder cellular signalling processes. Smaller NPs can be taken up through endocytosis, which could permeabilize endosomal membranes, interfere with transport carrier trafficking, and perturb endosomal membrane traffic. Released into the cytosol, NPs impact crucial organelles like the nucleus or mitochondria, also interfering with biological processes such as chromosomal migration during cell division and the formation of mitotic spindles. NPS also affects exocytosis by affecting the transport carriers. NPs interfere with endosomal membrane traffic by reducing signalling, altering surface protein turnover, and retrograde signalling [10]. The accumulation of NPs in late endosomes or lysosomes could impair the critical cellular membrane turnover process of macroautophagy, potentially leading to autophagic cell death. Internalised NPs may also stimulate autophagy. These processes contribute to stress responses at the plasma membrane and endo-lysosomes, triggering cellular stress, which triggers the generation of reactive oxygen species (ROS) and causes sterile inflammation. The intrinsic immune system, which encompasses Toll-like receptors (TLRs), can react to alarmins or damage-associated molecular patterns (DAMP), resulting in sterile inflammation [11].

### The health effects of NP

The major routes of entry for the NPs in the human body are inhalation, ingestion, and skin contact. Based on their size, shape, and surface properties, NPs gradually accumulate in the respiratory system, with the smaller ones deposited in the lower airways near the alveolar region, damaging lung cells. The deposition of particles in the respiratory system is dependent on the surface chemistry, with hydrophilic surfaces being less prone to deposition than hydrophobic particles. Elongated particles have a structural advantage for deposition in the respiratory system when compared to spherical particles. NPs that are accumulated in the respiratory system can interact with various pulmonary cells, including alveolar macrophages, epithelial cells, and fibroblasts. These cells can identify and engulf nanoparticles through phagocytosis or pinocytosis. The size of the particles, their surface chemistry, and the amount of exposure all have a significant impact on the cellular reactions to exposure to NPs [12].

The detrimental effects of NPs are primarily based on their physicochemical properties and exposure. For instance, animal studies have demonstrated that titanium dioxide nanoparticles (TiO2 NPs) can cause lung inflammation and fibrosis. Biomarkers have been used to investigate the correlation between NPs and their associated health effects [13]. Neuropeptides affect the endocrine system through various mechanisms. After entering the cell, nanoparticles attach to hormone receptors, which subsequently trigger or hinder the activation of signalling pathways further down the line, exerting a mimicking effect. For example, metallic nanoparticles, Ag ZnO, bind with oestrogen receptors to exert estrogenic effects. This disrupts endocrinal homeostasis, especially during pregnancy or childhood. Increasing the level of oxidative stress is another way of having a deleterious effect on the body. Nanoparticles produce reactive oxygen species (ROS) within cells, resulting in cellular damage and interference with signalling pathways.

Carbon-based NPs inhibit aromatase enzyme activity, thus interfering with oestrogen biosynthesis, which lowers oestrogen levels and leads to endocrine dysfunction. When zebrafish were exposed to TiO2 NPs in the presence of decabromodiphenyl ether-209 (BDE-209) and pentachlorophenol (PCP), the toxicity effect was greater, and it disrupted the thyroxin levels [14]. In vivo research on ZnO NPs showed that they act on Leydig cells to decrease the rate of steroidogenesis in mice [15]. More research is required to assess the possible hazards of different types of NPs causing pathological disruptions to the endocrine system. Administering Cd-NPs in mice caused immunodeficiency by abrupt reduction and viability of monocytes, along with lymphocyte transformation [16].

NPs affect the male reproductive system through DNA damage leading to mutation. The inflammatory response is evident in the testicular tissue, as NPs can cross the blood-testis barrier, causing oxidative stress and cellular death. All these cause oligospermia and male infertility. Au-NPs, Ag NPs, CNTs, SiO2 NPs, ZnO NPs, and CeO2 NPs could enter the testis, while Au-NPs, Ag-NPs, TiO2-NPs, and CeO2-NPs persistently accumulate in the testicular structures and damage them [17]. NPs can cross the placental barrier, thus entering the foetal blood stream, which may have long-term effects on the child.

### Carcinogenicity of NPs

Studies have demonstrated that NPs have carcinogenic properties. This detrimental impact is brought on by ROS-induced DNA damage, mutations, apoptosis, inhibition of the cell cycle, increased cytokine and chemokine secretion, inflammatory responses, immunosuppression, and decreased viability of major innate and adaptive immune system cell types.

There is a scarcity of publications on the carcinogenicity of NPs, which could be because cancer takes time to develop. Numerous variables, including size, shape, surface chemistry, and physicochemical characteristics, influence the intricate pathways that underlie the harmful impacts of NPs. The entire toxicological mechanism of NPs is currently unknown. Nonetheless, several investigations have demonstrated that NPs’ primary cause of toxicity is their capacity to produce ROS and cause oxidative stress. Highly reactive ROS can harm cells’ lipids, proteins, and DNA, resulting in cell death or malfunction. By stimulating the immune system and producing proinflammatory cytokines, NPs can potentially cause inflammation. [18]

### Conclusion:

The use of nanoparticles in medicine can address global public health concerns by overcoming challenges. NPs can play a crucial role in the diagnosis and treatment of a vast number of diseases because of their unique properties. NPs enhance drug bioavailability, targeting ability, and efficacy. Promising results of vaccine manufacturing from lipid nanoparticles pave the pathway for future research. Concomitantly, NPs pose a significant threat to the environment and human health since their accumulation increases progressively over time. Interruption of the signalling pathways, apoptosis, inflammatory response, ROS generation, DNA damage, and mutation are the major challenges with NPs. Concerns regarding acute toxicity or long-term consequences are unfounded. Further research may unfold to understand pathological mechanisms at the cellular and tissue levels. Collaborative efforts between ecologists and epidemiologists through intricate observance of the food chain are also required. The future lies in the rational use of nanomaterials while minimising harmful effects on human health, the environment, and society.
